# Alumina-Based Cutting Tools—A Review of Recent Progress

**DOI:** 10.3390/ma18122813

**Published:** 2025-06-16

**Authors:** Irena Žmak, Sonja Jozić, Lidija Ćurković, Tomislav Filetin

**Affiliations:** 1 Department of Materials, Faculty of Mechanical Engineering and Naval Architecture, University of Zagreb, 10000 Zagreb, Croatia; lidija.curkovic@fsb.unizg.hr (L.Ć.); tomislav.filetin@fsb.unizg.hr (T.F.); 2 Department of Manufacturing Engineering, Faculty of Electrical Engineering, Mechanical Engineering and Naval Architecture, University of Split, 21000 Split, Croatia; sonja.jozic@fesb.hr

**Keywords:** cutting tools, ceramics, alumina-based, cutting inserts, sintering, doping, sustainability, Sustainable Development Goals

## Abstract

Choosing the appropriate cutting tool material is essential for enhancing machining processes because it directly affects product quality, surface finish, dimensional accuracy, tool longevity, and overall efficiency. Different materials are used for cutting tools, i.e., for cutting inserts. Due to their high hardness and high temperature resistance, ceramics cutting inserts allow for increased cutting speeds, resulting in shorter manufacturing times and reduced costs, despite being pricier than traditional cemented carbide and facing certain technical challenges due to their brittleness. Alumina-based ceramics dominate the market, accounting for about two-thirds of usage, followed by silicon nitride and zirconia. This paper provides a comprehensive overview of recent advances in alumina ceramic materials used as cutting inserts, focusing on research conducted in the last five years to optimize static and dynamic mechanical and thermal properties, wear resistance, density, etc. They ways in which the properties are altered through the incorporation of whiskers, nanoparticles, or nanotubes; the modification of the structure; the optimization of sintering parameters; and the application of advanced sintering techniques are demonstrated. The paper also addresses sustainability, environmental impact, and the management of critical raw materials associated with cutting inserts, which pertains to the future development of cutting insert materials.

## 1. Introduction

Cutting tools are used to remove material from the workpiece during machining. Adequate material selection for cutting tool materials is essential in all machining processes, as it directly influences product quality, surface finish, dimensional accuracy, and aesthetics. It also impacts tool life, the machining process’s efficiency, and the balance between cost and efficiency. Selecting the right cutting tool material, sometimes with additional coatings and tool geometry, is needed when optimizing machining for a specific material.

Traditional materials used as cutters were hardened steels applied to cutting materials with lower hardness than themselves. During machining, a large amount of heat is generated due to high friction, which can decrease the hardness of the hardened steel, making it unsuitable for further use. Nowadays, the most widely used cutting tool materials are cemented carbide (containing tungsten carbide), high-speed steels, ceramics, cermets (containing carbides other than tungsten carbide, such as TiC, TiCN, and TiN in a metallic binder, like Ni, Mo, or Co), cubic boron nitride, and polycrystalline diamond (PCD). Developing new cutting tool materials aims to increase cutting speeds, improve machining efficiency, reduce wear by increasing high-temperature hardness, enhance fatigue resistance, and minimize environmental impact.

**Figure 1:**
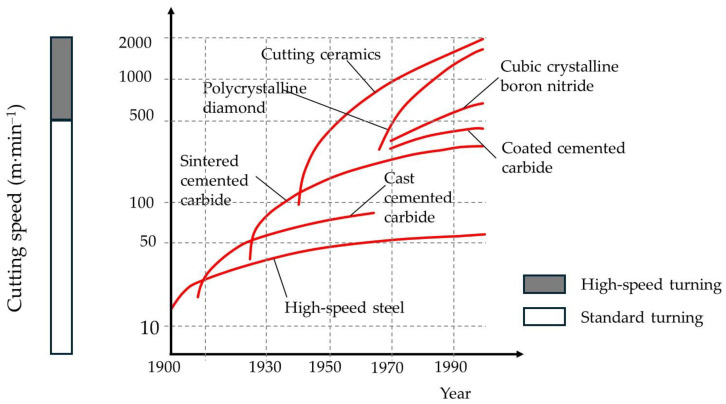
Advancements in cutting tool materials vs. cutting speeds (reproduced with permission from Wegener, K. et al., Procedia CIRP; published by Elsevier, 2016) [2].

About one hundred years ago, cemented carbides were invented and used as novel materials for machining hard materials at high cutting speeds. It is estimated that cemented carbide makes up over 80% of all cutting inserts in today’s industry [1]. A typical cemented carbide comprises approximately 90 wt.% ceramic components, with tungsten carbide being the most used, and 10 wt.% metal, typically, but not exclusively, cobalt. All components are prepared as powders, milled, compacted, and sintered. Similarly, ceramic cutting inserts were introduced after World War II, which allowed even greater cutting speeds and thermal resistance (Figure 1) [2]. The first ceramic used for this purpose was alumina, also known as aluminum oxide (Al_2_O_3_). Besides alumina, the most common ceramic inserts used nowadays are based on zirconia (zirconium oxide, ZrO_2_) or silicon nitride (Si_3_N_4_). Ceramic cutting inserts are generally several times more expensive than traditional cemented carbide inserts but offer significant advantages in specific applications.

In high-speed machining, ceramics can withstand much higher cutting speeds than cemented carbide, especially alumina, for hard materials like hardened steels, nickel-based superalloys [3], ductile iron, and cast iron [4]. Alumina offers good machining properties for high-strength manganese-rich stainless steel, bronze, brass, and cobalt superalloy [5].

Besides metals machining, similar problems arise in wood machining, where conventionally used cemented carbides are also being replaced by ceramic cutting inserts to address the difficulties of intricate wood structures with elements of greater hardness, such as knots, and the presence of abrasive components, such as sand from the soil [6].

Recent studies focus on replacing metallic binders with ceramic ones in tungsten carbide-based cutting inserts to develop cutting inserts with superior wear resistance. Metallic binders, such as cobalt, in cemented carbides impose a wear limit during high-speed cutting operations due to metal softening and are related to the problems of oxidative corrosion [7]. To enhance the tribological properties of WC-based cemented carbide cutting inserts, a ceramic composite powder comprising 20 wt.% alumina, 3.9 wt.% yttria, and the remainder zirconia were utilized to substitute the metallic binder. Compared to binderless, pure tungsten carbide ceramics, the wear rate decreased by 30% when 8 wt.% of ceramic composite powder was used. The lowest steady-state friction coefficient of 0.49 was recorded at 12 wt.% ceramics, compared to 0.75 at pure WC [8]. Incorporating VC and Cr_3_C_2_ effectively managed the size of WC grains and improved the uniform distribution of ZrO_2_ and Al_2_O_3_ grains within the WC matrix [9].

Unique advanced ceramics, such as polycrystalline cubic boron nitride (PcBN), also noted as PCBN or CNB, and polycrystalline diamond, have been developed for machining metals that are difficult to cut, such as titanium alloys [10]. Cubic boron nitride is often referred to as a superhard material due to its exceptional hardness, which ranks just below that of diamond. PcBN offers better thermal stability and reduced reactivity with iron than diamond [11]. Recently, PcBN composites have been investigated to improve PcBN further. A recent 2023 study focused on enhancing the cutting performance of the PcBN by incorporating yttria (Y_2_O_3_)-stabilized zirconia (ZrO_2_) by high-pressure sintering at 5.5 GPa and 1500 °C [12]. Applying the same process parameters, but over a slightly extended duration and adding 6 wt.% tungsten, improved overall PcBN performance, including cutting performance, hardness, wear, flexural strength, and density [13]. The optimal temperature was 1500 °C within the studied range of 1380 °C to 1560 °C for the TiN0.3/AlN/TaC/WC/VC PcBN composites sintered at 5.5 GPa, with a holding time of 10 min, resulting in a hardness of 26.0 GPa and a toughness of 8.8 MPa·√m, as published by Zou et al. in 2024 [11]. Impact loads present a challenge when cutting hardened steels, leading to a study aimed at enhancing the stability of PcBN tools by developing a PcBN composite reinforced with two layers of microfibers: one with silicon carbide whiskers and the other with alumina whiskers [14].

**Figure 2:**
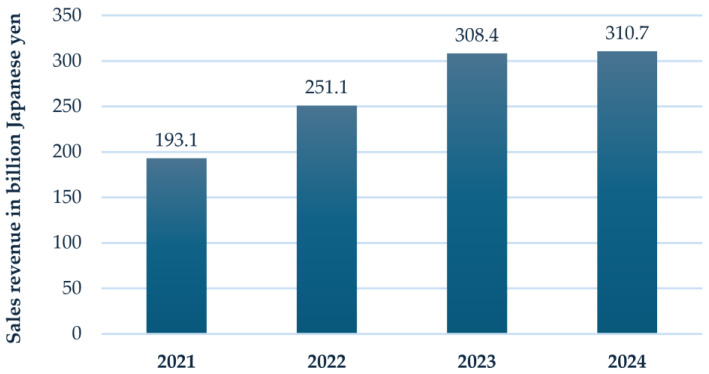
Sales revenue of Kyocera Corporation’s industrial tools segment from fiscal year 2021 to 2024 (data from Statista) [15].

Indeed, the cutting tools sector, composed of diverse, most advanced materials, has grown rapidly in recent years, as highlighted by the Japanese manufacturer Kyocera, by about 50% (Figure 2) [15].

To make the machining processes more environmentally friendly, there is an ongoing need to minimize unnecessary waste caused by cutting inserts’ short tool life, so creating new materials that last longer is essential. An estimate is that by increasing the total tool life by approximately 50% to 100%, the production cycle time could decrease by approximately 15%, and the energy consumption could be reduced by 12% [16]. More durable alumina tools reduce the raw materials needed for tool production. Consequently, this represents a step closer to the objective of sustainably using natural resources, as stated in Goal Number 12: “Ensure Sustainable Consumption and Production Patterns” of the United Nations’ Sustainable Development Goals (SDGs). This goal seeks to ensure sustainable management and efficient use of natural resources by 2030 [17].

Cobalt is the most common binder metal in tungsten carbide cutting inserts, but it raises several sustainability concerns. These include limited recycling and recovery options, economic fluctuations, the environmental impact of extraction, social issues, and vulnerabilities in the geopolitical supply chain. Since 2011, cobalt and tungsten have been listed as critical raw materials (CRM) in the European Union due to the elevated risks of supply shortages and their economic effects compared to most other raw materials [18]. The challenge of critical raw materials demands a scientific approach through various simultaneous actions. These include enhancing production processes, identifying suitable substitutes for partial or complete replacement, and enhancing recycling efforts [19].

It is also worth noting that bauxite was included in the European Union’s fourth published list of critical raw materials in 2020 [20], primarily due to its use in producing aluminum metal and alloys. Bauxite ore is transformed into alumina through the Bayer process, and each ton of produced alumina yields between 1 and 1.5 tons of high-alkaline and environmentally challenging waste known as red mud [21]. Sustainable options for red mud waste involve utilizing it in cement production, which reduces emissions, conserves raw materials, and enhances the properties of the cement [22,23].

With the increasing emphasis on sustainability, recent research analyzed the use of natural rocks as cutting tools, such as alta quartzite, basalt, Brazil quartzite, flint garnet, lamellar obsidian, porphyry, quartz, silver quartzite, and wine-red quartzite [24]. Proposed performance improvements for rock inserts focus on grinding and better forming the cutting-edge roundings [25]. Further studies examined the effectiveness of natural rocks as cutting inserts when coated with titanium nitride (TiN) via physical vapor deposition (PVD) [26].

Additionally, liquid coolants typically involve mineral oils and organic additives, such as corrosion inhibitors, emulsifiers, and lubricants, thus making dry machining using ceramic cutting inserts a much more sustainable choice. Promoting a safer working environment is connected to Sustainable Development Goal Number 8: “Promote sustained, inclusive and sustainable economic growth, full and productive employment and decent work for all” [17]. Self-lubricating ceramic cutting inserts enhance tool performance and decrease manufacturing costs [27,28]. Another way to make dry machining with ceramic cutting inserts even more environmentally friendly is to use cryogenic coolants [29]. The most developed cryogenic machining method is the cryogenic spray method, where liquefied CO_2_ extends tool life by lowering the cutting forces by 60% compared to liquid coolants [30]. On the other hand, achieving reliable dry machining is challenging due to modern tools’ wide application range and complex geometries [31].

Another way to extend the life of ceramic cutting tools is to enhance cooling in the cutting zone by modifying the tool profile to include an internal cooling system in the insert [32]. This is feasible when ceramic inserts are produced through additive manufacturing instead of molding [33].

Research on non-conventional ceramic manufacturing methods involves developing novel techniques for preparing ceramic green bodies and advanced technologies for ceramic sintering. These efforts toward more sustainable machining are encompassed in Sustainable Development Goal Number 9: “Industry, Innovation, and Infrastructure”, which focuses on fostering industrial innovation [17]. Reducing the sintering time and/or temperature is also relevant when developing new sintering processes to improve specific properties or achieve a particular structure. The reduction in energy needed for sintering is directly connected to the target of sustained greenhouse gas emission reduction, which is associated with SDG No. 13, “Take urgent action to combat climate change and its impacts” [17]. Similarly, reducing energy use in machining by enhancing energy efficiency improves sustainability.

The basic concept of removing material through cutting applies to conventional machining processes, including milling, turning, drilling, and grinding. There are many similarities in material selection, effectiveness, heat dissipation, hardness, wear resistance, and more [34]. Alumina is among the most widely used abrasive materials in grinding wheels. This manuscript does not deal with alumina ceramics for grinding tools but instead focus solely on cutting tools with determined geometry made of bulk alumina-based ceramics with specific edge geometries.

The research field of ceramics for cutting inserts is broad, presenting numerous opportunities for enhancements aimed at a more sustainable future. This brief review focuses on significant advancements in composition, properties, and selected processing within the subfield of alumina-based ceramic tools over the last five years.

## 2. Alumina as Cutting Tool Inserts

Cutting inserts are specialized and interchangeable components that are part of a modular cutting tool. Alumina (Al_2_O_3_) is a technical ceramic widely utilized for cutting inserts due to its high hardness, wear resistance, thermal stability, and chemical resistance. Due to high hot hardness (retaining high hardness at high temperatures) and chemical resistance in contact with ferrous materials, alumina is especially appropriate for finishing hardened steels, hard alloyed steels, and hard cast iron. High hot hardness eliminates the need for liquid coolants, enabling high-speed dry machining in situations where cooling is challenging or undesirable, such as in boring operations. Alumina is particularly well-suited for finishing hardened components in the automotive industry, such as gears, bearings, and shafts, as well as in the manufacturing of tools and dies. It is frequently employed to achieve an excellent surface finish after utilizing more economical cemented carbide inserts, thereby enabling the achievement of improved required tolerances. The enhancement of alumina’s toughness through the incorporation of zirconia or the reinforcement with silicon carbide whiskers enables its effective application in machining nickel-based superalloys, which are used in gas turbines within the aerospace and energy sectors. Minimal tool wear and extended tool life under stable thermal and mechanical loads render alumina ideal for cutting inserts utilized in mass production with consistent cutting conditions. Alumina performs very well under dry machining, eliminating the need for lubricants, which represents a more environmentally friendly and sustainable option that contributes to the principles of the circular economy.

Compared to cemented carbide, ceramics used as cutting inserts are more heat-resistant and maintain high hardness at high temperatures that develop due to high friction forces during cutting, thereby reducing the need for coolants. The excellent wear resistance of ceramics extends tool life, making them a reliable choice for extensive and continuous machining. Some ceramic materials, such as alumina, are relatively brittle, so they work best in non-interrupted cuts [35]. Pure alumina cutting inserts are also sensitive to vibrations and are prone to chipping and breaking under interrupted cutting conditions. A better choice of ceramic inserts for interrupted cutting is silicon nitride, which has slightly lower hardness and wear resistance than alumina but offers much better toughness (Table 1) [36].

**Figure 3:**
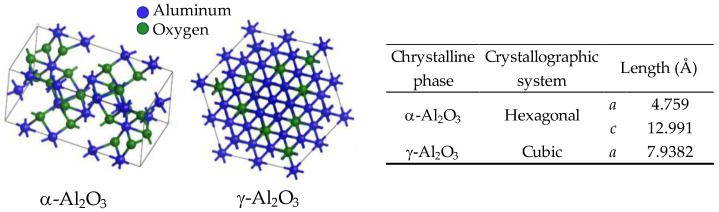
Crystal structures of α and γ alumina phases (adapted and reproduced with permission from Silva-Holguín, P.N. et al., Materials; published by MDPI, 2022) [42].

Alumina has a single thermodynamically stable alpha (α) phase, and it is the preferred phase for structural ceramics components, such as cutting inserts, as it features the most compact crystal structure [37]. The polycrystalline α-phase in wear-resistant cutting inserts alumina contributes to thermal stability. The α-phase features a hexagonal close-packed (hcp) structure, where two-thirds of the octahedral interstices are filled with Al^3+^ cations. Alumina also occurs in various metastable phases, such as gamma (γ) (Figure 3), delta (δ), theta (θ), eta (η), and kappa (κ), with some serving roles as catalytic carrier materials and catalysts [38]. Heating alumina raw materials like boehmite (AlO(OH)), found in the ore bauxite, under controlled conditions, generates different alumina phases depending on temperature, with the stable α-phase forming at about 1150 °C.

Lowering the heating rate can control phase transformations during sintering. A lower heating rate influences the rearrangement of grains during the transformation from θ to α-alumina (Al_2_O_3_), thus enhancing the density and avoiding the formation of a vermicular structure [39].

However, a calcination temperature exceeding 1400 °C for commercial alumina is essential for fully converting to the α-Al_2_O_3_ phase [40], i.e., a pre-sintering process is essential for Al_2_O_3_ powder to minimize shrinkage and enhance product quality. A recent study by Zhou et al. [41] calculated the solid-state temperature–pressure phase diagram using the first-principles quasiharmonic approach for four alumina phases: α, γ, κ, and θ, which could be used to synthesize different alumina phases experimentally (Figure 3) [41].

Because alumina ceramic cutting inserts are more expensive, they are generally used when their benefits compensate for the costs, for example, when cemented carbide inserts wear out too fast. Indeed, using alumina-based cutting inserts is far more affordable than using PcBN, which is commonly about 72% [43].

Alumina-based cutting inserts can be used in dry-cutting conditions; unlike high-speed steel tools and cemented carbides, alumina does not require coolant. This enables a more environmentally friendly machining process [44], reduces occupational risks for operators, eliminates the need for a drainage system, and lowers the machining costs [45].

### 2.1. Doped vs. Composite Ceramics—Not to Be Confused with CMCs

Dopants are compounds added in low quantities (typically below 5 wt.%) to ceramics to enhance their properties without changing the structure. Dopants integrate into the base material, affecting its properties at an atomic level. In contrast to single-phase ceramics, composite ceramics are a mixture of two or more ceramic compounds (typically above 5 wt.%) that do not fully dissolve and are combined to tailor the properties of the composite material.

Composite ceramics should be distinguished from the well-known ceramic matrix composites (CMCs). Composite ceramics have various ceramic phases without a defined matrix-reinforcement structure. In contrast, CMCs consist of a ceramic matrix reinforced with ceramic fibers or particles, distinguishing between the matrix and the reinforcement. Composite ceramics are primarily used to manufacture advanced cutting inserts and wear-resistant coatings. On the other hand, ceramic matrix composites are designed mainly for high-temperature components used in automotive and aerospace applications for heat shielding. One example of novel alumina-based CMCs is the alumina-chromium composites sintered at 1400 °C using electron beam melting (EBM), in which the thermal and electrical conductivity can be adjusted by altering the chromium content [46].

### 2.2. Doped Alumina for Cutting Inserts

Due to numerous and diverse ceramic manufacturing processes, various additives are studied concerning distinct manufacturing and sintering processes. Boldin et al. investigated the densification through different stages of spark plasma sintering of alumina-based ceramics containing three different additives: 0.5 vol.% MgO, then 0.5 vol.% TiO_2_, and lastly, 0.5 vol.% ZrO_2_ added to α-Al_2_O_3_ [47]. In the early stages of sintering, i.e., up to about 70% density, the densification occurs as necks form between alumina particles, and the additives have a minimal impact on the densification. The second sintering stage, from approximately 70% to 90% relative density, involves a process of intensive densification, during which a complex open porosity develops. In the second phase, particles of MgO, TiO_2_, and ZrO_2_ situated at alumina grain boundaries do not participate in grain boundary sliding. The additives were determined to influence the densification solely during the third and final stage of spark plasma sintering, i.e., from 90% to approximately 100% relative density: magnesia and zirconia inhibit grain growth, whereas titania increases it. The addition of titania improved crack resistance, presumably due to the intergranular fracture motion caused by the clusters of the formed second phase [47].

Doping of 400 ppm lanthanide (Ln^3+^) in the alumina lattice significantly postpones the beginning of phase transformation θ → α. It is suggested that Ln-dopants occupy the empty octahedral sites in the alumina lattice [48].

#### 2.2.1. Doping Alumina with Titania

Titania (or titanium oxide, TiO_2_) has a lower melting temperature (*T*_m_ = 1840 °C, [49]) than alumina (*T*_m_ = 2041 °C, [50]) and it efficiently binds alumina powder particles during sintering, which results in less porosity, i.e., increased density and enhanced wear resistance [51]. When alumina is doped with titania, its hardness and fracture toughness improve.

**Figure 4:**
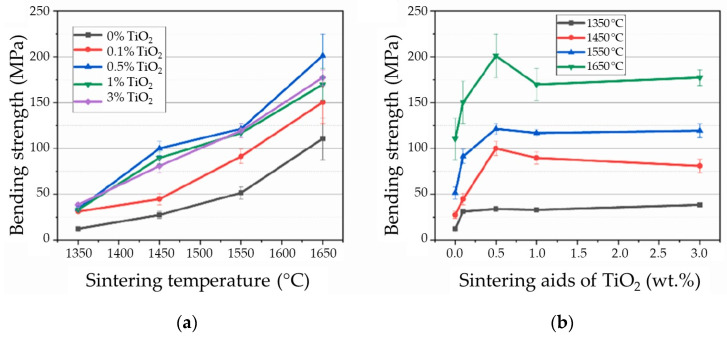
Bending strength of alumina ceramics doped with titania and formed by stereolithography: (**a**) Influence of sintering temperature; (**b**) Influence of content of TiO_2_ (reproduced with permission from Qian, C. et al., Ceramics International; published by Elsevier, 2023) [52].

A recent study by Qian et al. focused on alumina ceramics produced through stereolithography and the incorporation of sintering aids, such as TiO_2_, ZrO_2_, CaO, and La_2_O_3_ [52]. Considering TiO_2_, the study examined how the addition of 0, 0.1 wt.%, 0.5 wt.%, 1 wt.%, and 3 wt.% affect bending strength, shrinkage, microstructure, and properties at different sintering temperatures. A 0.5% addition of TiO_2_ significantly improved the bending strength of alumina, raising it from 110.4 MPa to 201.1 MPa at a sintering temperature of 1650 °C (Figure 4) because of the development of a moderate amount of the intergranular Al_2_TiO_5_ s phase. Furthermore, the grains were most uniform at these parameters and had the highest density. The rapid grain growth observed in ceramics produced through stereolithography resembles that seen in ceramics formed by traditional methods after the addition of TiO_2_ during sintering.

Earlier studies indicate that TiO_2_ enhances the alumina properties due to increased diffusion rate when sintering alumina by substituting Ti^4+^ for Al^3+^, leading to a higher concentration of Al^3+^ vacancies. On the other hand, recent studies find that the differences in thermal expansion coefficients between Al_2_TiO_5_ and Al_2_O_3_ cause a decrease in bending strength when more than 0.5% TiO_2_ is added to improve alumina [52].

Titania-alumina composites are primarily used when durable, wear-resistant coatings are needed, along with excellent corrosion resistance and thermal stability [53], longer tool life when cutting hard or abrasive materials, lower friction on the flank rake, and potential for higher cutting speeds [54].

Haldar et al. found that the highest relative density of 98.25% was achieved when 1 wt.% of nano-sized TiO_2_ was added to alumina before sintering at 1600 °C [55]. The excess of TiO_2_ resulted in the secondary phase formation at grain boundaries: low-density Al_2_TiO_5_, which does not significantly influence the densification. What was also noted was the increase in fracture toughness and the reduction in friction coefficient. Hardness improved by 8.82%, while the specific wear rate increased by 45.56%. The morphology of the sintered Al_2_O_3_ was significantly modified, too. Wear depth decreased, and a combination of plastic deformation and abrasion mainly caused wear [55].

Ceramics are sensitive to thermal shock failure due to their low toughness, poor thermal conductivity, high Young’s modulus, and elevated thermal expansion coefficient [56]. Thermal shock resistance of ceramics depends on the fracture strength (*σ_f_*), Young’s modulus (*E*), thermal expansion coefficient (*α*), and thermal conductivity coefficient (*k*) [57]. It can be estimated by the thermal shock resistance parameter (*R*′), i.e., the maximum temperature difference (Δ*T_C_*) a ceramic can endure before a crack starts to form:

(1) $$R^{\prime }=∆\;T_{C}=\frac{\sigma_{f\;}(1-\nu )}{\alpha \;E}k$$

where *ν* is Poisson’s ratio. It has been shown that the lifespan of ceramic cutting inserts is affected by thermal shock cracking, together with mechanical shock [58]. Thus, the greater the thermal conductivity coefficient of the ceramic cutting insert, the better. As explained above, when TiO_2_ is added to alumina, various important properties increase, namely density, wear resistance, hardness, and fracture toughness, while the friction coefficient decreases.

**Figure 5:**
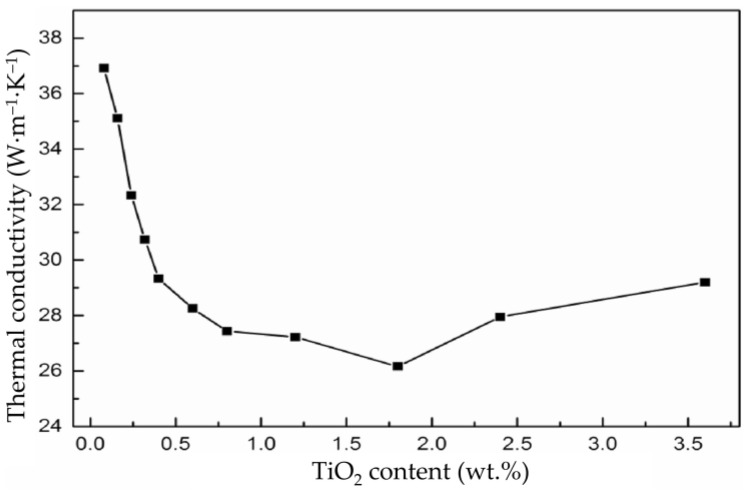
Thermal conductivity coefficient vs. added titania to alumina (reproduced with permission from Zhou, N. et al., Ceramics International; published by Elsevier, 2024) [59].

Conversely, the addition of TiO_2_ negatively affects the thermal shock resistance of alumina. For example, a recent study investigated how the thermal conductivity of alumina changes with different amounts of added titania [59]. This research, carried out by Zhou et al., indicated that when alumina, with the addition of titania from 0.08 wt.% to 3.6 wt.%, was sintered in a reducing atmosphere at 1500 °C, the increased amount of titania reduced the ceramics’ heat conductivity by roughly a third, i.e., from approximately 37 W·m^−1^·K^−1^ to approximately 26 W·m^−1^·K^−1^ with adding TiO_2_ up to 1.75 wt.%, after which the heat conductivity coefficient began to increase gradually (Figure 5) [59]. According to Equation (1), a thermal conductivity coefficient reduced by a third reduces ceramics’ thermal shock resistance parameter by a third, i.e., significantly decreases this important property when using ceramics as cutting inserts. This illustrates how one addition can provide numerous benefits while potentially harming another property. Additional investigation is needed to tackle such possible emerging drawbacks.

It is also worth noting that ceramic cutting inserts have considerably lower thermal conductivity and thermal shock resistance than cemented carbide cutting inserts, which have metallic cobalt binders.

#### 2.2.2. Manganese Oxide Doped Alumina

In addition to titania, manganese oxide, such as 0.5 wt.% of MnO, can be included as a co-dopant to enhance density, microhardness, and compressive strength. Manganese also influences the transparency of alumina, which is not essential for cutting tool inserts but can be advantageous in other application areas [60]. During sintering, manganese oxide nanoparticles dissolve, enabling manganese atoms to replace aluminum atoms at grain boundaries and within bulk alumina. This secondary phase suppresses grain growth and improves mechanical properties [61]. In another recent study, it was found that nanoparticles MnAl_2_O_4_, found at grain boundaries, decreased Young’s modulus to 370 GPa ± 5 GPa compared to 400 GPa ± 5 GPa for pure alumina; hardness was not affected, and the fracture toughness increased by 23% [62].

**Figure 6:**
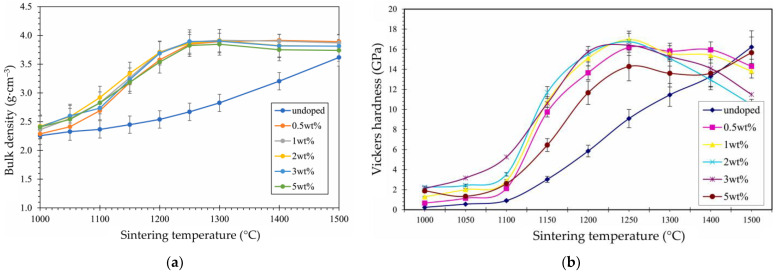
Effect of co-doping alumina with different total titania and manganese oxide amounts, equally balanced: (**a**) Sintering temperature vs. bulk density; (**b**) Sintering temperature vs. Vickers hardness (reproduced with permission from Gnanasagaran, C.L. et al., Ceramics International; published by Elsevier, 2022) [63].

A recent research gap was identified in combining dopants, titania, and manganese oxide to enhance the properties of alumina. Hybrid doping in different amounts has shown that bulk density is significantly improved compared to undoped alumina at sintering temperatures between 1000 °C and 1400 °C (Figure 6a). For a high total amount of dopants (5.0 wt.%), relative density decreased when the sintering temperature increased at and above 1400 °C, which was reflected in the decrease in Young’s modulus and Vickers hardness (Figure 6b) [63]. It was demonstrated that the porosity decreased by approximately 40% when up to 3 wt.% of titania and manganese oxide was used. However, the density was reduced for larger amounts due to what is presumed to be dopant agglomeration [63].

**Figure 7:**
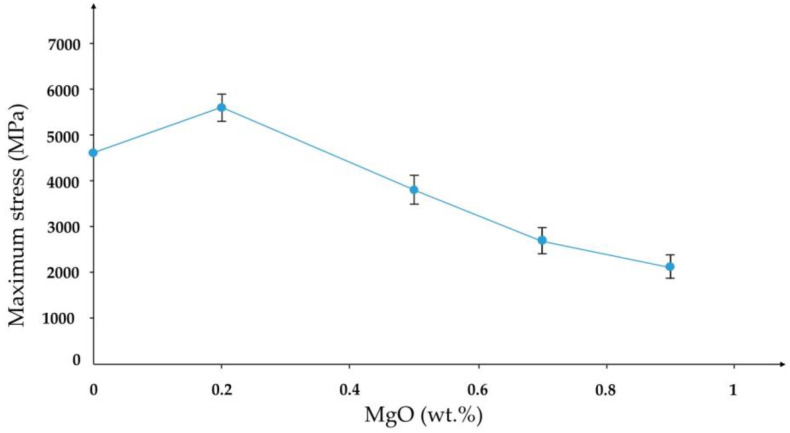
Maximum dynamic compressive stress of ZTA with varying amounts of MgO (reproduced with permission from Arab, A. et al., Materials; published by MDPI, 2019) [65].

Studies on the behavior of brittle materials, such as ceramics, are limited, and the mechanisms of failure remain unclear because ceramics exhibit a more complex mechanical response than metals, especially when subjected to multiaxial stresses. The ceramic’s dynamic compressive failure stress relies on several factors, including grain size, porosity, and the density of microcracks. Metals primarily undergo plastic deformation and strain hardening to absorb kinetic energy. At the same time, stress-transfer mechanisms and possible localized brittle fracture influence the dynamic behavior of ceramics, leading to their abrupt failure [64]. A study on adding TiO_2_ to alumina showed that adding more than 0.2 wt.% of MgO reduced the dynamic compressive strength (Figure 7). The addition of only 0.2 wt.% MgO to the Zirconia-Toughened Alumina (ZTA) reduced grain size and porosity, thus leading to an increase in dynamic failure strength [65].

### 2.3. Zirconia-Toughened Alumina (ZTA)

Cutting inserts based on reinforced alumina were developed for high-speed machining. The production of such ceramic matrix composites aims to achieve even higher thermal stability and mechanical properties compared to unreinforced alumina [66]. Alumina’s most prevalent reinforcements are zirconia (ZrO_2_) [67], titania (TiO_2_), titanium carbide (TiC), silicon carbide (SiC), zirconium carbide (ZrC) [68,69], titanium diboride (TiB_2_), polycrystalline cubic boron nitride (PcBN), etc. When multiple types of reinforcement are used, they are referred to as hybrid composites [70]. Particles, whiskers, and fibers are commonly used as reinforcements. Platelet reinforcements can be added directly to the mixture before or during sintering (in situ) through chemical compounds that produce platelets.

Alumina is known for its high hardness, hence its high abrasion resistance. It is a stiff ceramic with a high Young’s modulus of theoretically up to 400 GPa but a lower strength and toughness of approximately 3.56 MPa√m [71]. When zirconia is added to alumina, these properties improve through microcracking, transformation toughening, and residual stress mechanisms. It was found that the minimum zirconia concentration required to prevent grain growth is 7 vol.% [72]. Due to their advantageous properties, zirconia-alumina ceramic composites have emerged as a leading choice for dry-machining ceramic cutting inserts. While Al_2_O_3_ exhibits lower fracture toughness, tetragonally stabilized ZrO_2_ demonstrates improved toughness up to 5 MPa√m [73].

The main factors influencing the grain growth of alumina ceramics include primary grain size, additives, and sintering methods and parameters. Adding nano alumina during pressureless sintering of alumina leads to a slight reduction in the grain size and a more uniform grain distribution. In the recent study by Zhao et al. [74], 1 wt.% nano alumina and 15 wt.% nano zirconia were used to study the alumina grain growth in ZTA when sintering by pressureless sintering [74]. Zirconia aligned along the alumina grain boundaries during sintering at 1600 °C for 1 h, inhibiting grain growth and reducing the average grain size from 11.73 μm to 5.63 μm. Most zirconia grains were found between the corners of alumina grains, and the amount of zirconia enclosed within alumina grains was low, enabling an effective grain growth inhibition [74]. A study of ZTA showed the highest abrasion wear resistance when 30 wt.% ZrO_2_ was used. Furthermore, introducing nano-dispersed zirconia to the same ZTA resulted in a 50% increase in its abrasion wear resistance [75].

Novel sintering methods are being studied to increase the wear resistance of alumina-based cutting inserts. The distribution of composite particles in composite ceramics affects mechanical properties, with intragranular structures generally outperforming intergranular ones. Intragranular structure changes the fracture mode of alumina grains from intergranular fracture to transgranular fracture [76]. Typical manufacturing methods rarely yield ceramics with an intragranular structure. Verma et al. have shown that innovative sol-gel-based processing can produce heterogeneous nucleation of the alumina matrix around well-distributed particles of the tetragonal zirconia as the second phase during calcination and sintering [77].

Different additives, or dopants, enhance the fracture toughness of zirconia-toughened alumina (ZTA) and make it more suitable for use as cutting inserts. Some are oxides: TiO_2_ [78,79], MgO, chromia (Cr_2_O_3_) [80], niobium pentoxide (Nb_2_O_5_) [81,82], CeO_2_, MnO_2_; others are carbonates, like strontianite (strontium carbonate, SrCO_3_), CaCO_3_ [83], etc. The use of graphene, a two-dimensional carbon nanomaterial, improves the flexural strength and toughness of alumina-based ceramic composites [84] the structural stability of graphene might be compromised due to high sintering temperatures or prolonged sintering times.

#### 2.3.1. Yttria Tetragonal Stabilized Zirconia for ZTA

**Figure 8:**
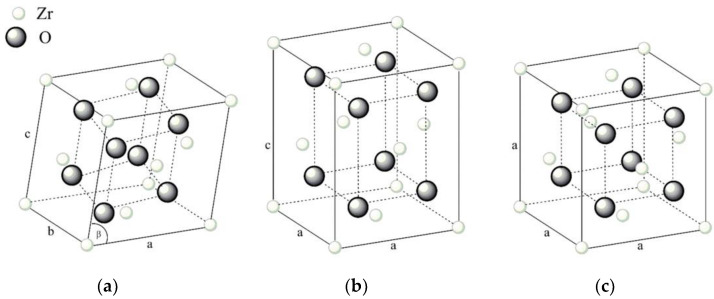
Zirconia crystal structures: (**a**) Monoclinic; (**b**) Tetragonal; (**c**) Cubic (Reproduced with permission from Sorrentino, R. et al., Materials; published by MDPI, 2019) [85].

Zirconia is a metastable allotropic chemical compound that can exist in various crystallographic structures at different temperatures. Zirconia naturally exists in the monoclinic crystal phase at room temperature (Figure 8a), where *a*, *b*, *c*, and *β* represent the lattice parameters [85]. The lattice transforms into the tetragonal crystal phase above 1170 °C (Figure 8b). Above 2370 °C, one more transformation occurs: into the cubic phase (Figure 8c) before ultimately melting at 2715 °C.

The molar volume of the monoclinic phase is larger than that of the tetragonal phase, so when this phase transformation occurs, a volume decrease of about 3% to 5% is expected [86]. The change in volume during this martensitic transformation results in a transformation strain of 4% to 5% and significant shear strains of 14% to 15% (or 9°), ultimately leading to the disintegration of sintered undoped zirconia grains [87]. If additional heating is applied, a comparable but slighter effect of increasing the volume occurs when zirconia transitions from a tetragonal to a cubic lattice. To maintain the tetragonal phase as a stable form at room temperature in zirconia, 3 mol.% yttria (Y_2_O_3_) as the dopant is commonly used. Yttria-stabilized zirconia (YSZ), more often abbreviated as 3Y-TZP (3% yttria-tetragonal zirconia polycrystal), is a common toughening agent in alumina composites widely used as cutting inserts [88].

#### 2.3.2. Magnesia Tetragonal Stabilized Zirconia for ZTA

Depending on the grain size, added dopants, applied sintering technique, and sintering parameters, toughness may considerably increase, for example, up to 10 MPa√m in magnesia (magnesium oxide) partially stabilized zirconia [89]. The mechanical properties, most notably the composite ceramic’s hardness, toughness, and wear resistance, are significantly influenced by its density, which depends on the powder preparation methods, sintering method, and sintering parameters [90]. This has allowed for the tailoring of mechanical properties, especially the fracture toughness of zirconia-alumina composites [91].

Compared to hot isostatic pressing, the slip casting technique is more cost-effective, although creating stable, concentrated suspensions for casting in molds can be challenging. Additives, such as ammonium polymethylacrylic acid, must be used to stabilize the water-based suspension during drying in the mold. Magnesium oxide (MgAl_2_O_4_), i.e., magnesium aluminate spinel, may be added to prevent irregular alumina grain growth during sintering. When limited amounts (up to 10 wt.%) of alumina were replaced with different amounts of tetragonal phase zirconia (t-ZrO_2_), hardness decreased by about 45%, while toughness doubled. A similar study [92] found that the longest extended tool life of alumina-zirconia ceramics was approximately 2.5 min at a cutting speed of 200 m·min^−1^. The ceramic tool’s primary wear mechanism involved notch and flank wear during the initial machining stage. In contrast, the dominant mechanism in the final stages was the formation of the built-up edge.

### 2.4. Molybdenum Reinforced Zirconia-Toughened Alumina

Zirconia-toughened alumina (ZTA) reinforced with molybdenum is a composite ceramic that is an essential type of self-lubricating cutting tool ceramic [93]. They are used where low friction and minimal wear are needed at elevated temperatures [94]. In addition to cutting tools, they are used for manufacturing dies and seals [95]. Ghosh et al. studied the tribological behavior of pressureless sintered ZTA at 500 °C and 750 °C for different molybdenum contents: 0, 5, 10, and 15, all wt.% [96]. Self-lubricating molybdenum oxides formed at tribological interfaces as the temperature rose from 500 °C to 750 °C. The lowest friction coefficient of 0.34 was observed at 750 °C for a 15 wt.% addition of molybdenum. The best wear resistance was achieved when 10 wt.% of molybdenum was added [96]. A former study by Qi et al. on molybdenum laminated alumina composites has found the formation of two new phases (MoO_3_ and MoO_2.8_) on the worn surface after the tribological testing at 800 °C, which points to metal molybdenum oxidizing at high temperatures through the reaction:

2Mo + 3O_2_ → 2MoO_3_ (2)

where the compound MoO_3_ formed on the worn surface serves as a lubricating film [97].

The addition of 10 wt.% molybdenum to zirconia toughened alumina sintered by pressureless sintering improved 5% to 10% the fracture toughness in comparison to monolithic ZTA [98].

### 2.5. Strontium Reinforced Zirconia-Toughened Alumina

Thakur et al. have studied a novel example of research: strontium carbonate (SrCO_3_), which forms strontium hexaluminate (SrO·6Al_2_O_3_) when added to alumina. Strontium is a promising alternative for generating platelets, using SrCO_3_ as the precursor to synthesize SrAl_12_O_19_ through the following reaction [6]:

SrCO_3_ + 6∙Al_2_O_3_ → SrAl_12_O_19_ + CO_2_ (3)

This compound has demonstrated the potential to improve the mechanical properties of alumina-based ceramics, yet its role remains limited. The specific crystal structure of SrO·6Al_2_O_3_ stimulates the growth of hexagonal platelets in alumina ceramics during its sintering. When SrCO_3_ was added to form in situ, theoretically, up to 7.5 wt.% of SrAl_12_O_19_ in a ZTA composed of 80 wt.% Al_2_O_3_ and 20 wt.% ZrO_2_, then HIP-ed (hot isostatic pressing, HIP) at 1350 °C and sintered at 1600 °C, high densities, and best properties were achieved at 5 wt.% of SrAl_12_O_19_ (Table 2) [6].

**Figure 9:**
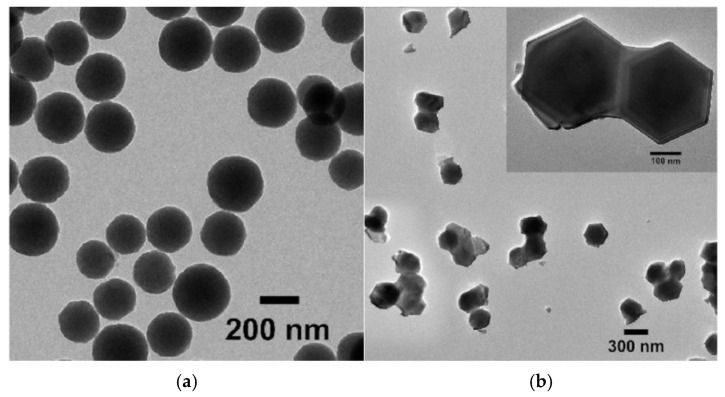
TEM micrographs of SrAl_12_O_19_ nanoparticles: (**a**) Precursor; (**b**) Annealed at 1150 °C (reproduced with permission from Afshani, J. et al., Journal of Luminescence; published by Elsevier, 2022) [99].

In addition to platelets forming in situ during sintering, nanoplatelets can be directly added to the mixture before forming the ceramic green body. Transmission electron microscopy (TEM) images of europium-doped SrAl_12_O_19_ precursor revealed spherical-shaped particles (Figure 9a). After annealing at 1150 °C, the nanoparticles transformed into a hexagonal shape (Figure 9b). The annealing process resulted in minimal particle aggregation, keeping the grain size from 200 nm to 300 nm in the nano range [99].

### 2.6. Multi-Walled Carbon Nanotubes (MWCNTs)-Alumina Hybrid Composites

Multi-walled carbon nanotubes (MWCNTs) were used to reinforce zirconia-magnesia alumina-based ceramic cutting inserts. The composite ceramics improved performance by ~27.6%, especially in the 200 m·min^−1^ to 300 m·min^−1^ cutting speed range, during continuous dry high-speed turning of hardened AISI-4340 steel with a hardness of ~40 HRC. The ZTA/MgO/MWCNT tools exhibit enhanced microhardness, nano-hardness, and indentation fracture toughness, resulting in improved cutting performance, especially under high cutting speeds, low feed rates, and shallow cutting depths. These tools exhibited outstanding resistance to three-body abrasive wear and fracture due to crack arrest and bridging fracture mechanisms. The composite demonstrated a significant improvement in wear resistance, with specific wear rates decreasing by approximately 66.07% and the coefficient of friction lowered by about 32.6%. Analysis using field emission scanning electron microscopy (FESEM) on worn specimens revealed a self-lubricating effect at the interfaces, shaped by the rolling and breaking actions of MWCNTs [100].

Incorporating MWCNTs into alumina effectively enhances various properties of nanocomposites, including thermal stability, electrical conductivity, Young’s modulus, fracture toughness, and strength. A recent study conducted by Toor and Shifa MWCNTs-alumina nanocomposites were fabricated using a novel combination of gas purging sonication, a technique used to enhance the dispersion of nanoparticles, and pressureless sintering [101]. Compared to pure alumina, the highest enhancement in mechanical properties was achieved at 1 wt.% of MWCNTs: Young’s modulus was 7.2%, fracture toughness 22%, and flexural strength 20.2% higher.

**Figure 10:**
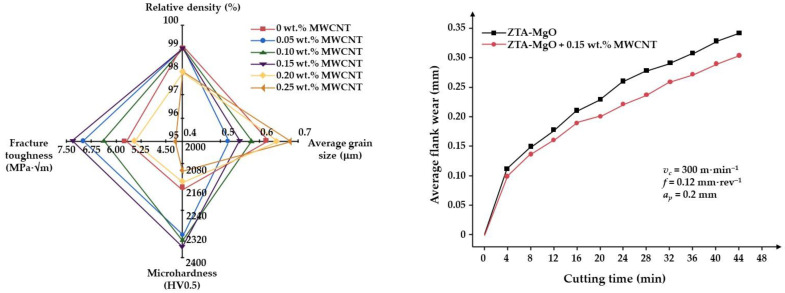
Influence of MWCNTs on mechanical properties and cutting inserts machining performance of hot-pressed ZTA-MgO ceramic (reproduced with permission from Prajapati, P.K. et al., Diamond and Related Materials; published by Elsevier, 2023) [102].

In a recent study, hot-press sintering at 1500 °C was employed to compact ZTA-MgO and 0–0.25 wt.% MWCNT powders [102]. The samples had relative densities ranging from 98% to 99%. The mechanical properties showed enhancement up to 0.15 wt.% of MWCNTs: hardness 2366 HV0.5 due to smaller grain size involving magnesium aluminum spinel (MgAl_2_O_4_) and MWCNTs, and fracture toughness at around 7.33 MPa·√m. Sintered samples with up to 0.15 wt.% MWCNTs were shaped as standard cutting tool geometry to be tested as cutting inserts in machining an AISI 4340 steel. The lowest cutting force with a high surface roughness was determined at 300 m·min^−1^·cutting speed (*v_c_*), 0.12 mm·rev^−1^·feed rate (*f*), and 0.2 mm depth of cut (*a_p_*) (Figure 10).

Alumina cutting inserts reinforced with MWCNTs have significantly decreased friction and improved thermal control, reducing the need for lubricant when used with nanofluids in minimum quantity lubrication (MQL) machining systems, i.e., the ones using less than 100 mL·h^−1^) for turning, drilling, milling, and grinding, resulting in better surface finish, as well as cutting tools’ extended durability [103,104].

A recent extensive review of hybrid MWCNTs–reinforced composites concluded that an overview of MWCNTs-doped ceramics has not been published yet. A review addressing current developments in multi-walled carbon nanotubes (MWCNTs) ceramic matrix composites, focusing on their structure, properties, and fundamental mechanisms, would be useful, as research into their application in ceramics is limited, presenting a potential focal point for future study [105]

### 2.7. Ceramic Matrix Composite with SiC Whiskers

Alumina reinforced with silicon carbide whiskers (Al_2_O_3_-SiC_w_) is a well-known and widely used ceramic matrix composite material for high-performance cutting tools. SiC whiskers are discontinuous fibers, like rods or needles, with diameters ranging from 0.1 μm to 1 μm and lengths from 5 μm to 100 μm. Alumina, which is reinforced with SiC whiskers, is the strongest and has the highest resistance to thermal shock among all Al_2_O_3_-based ceramics. Alumina matrix provides high hardness and chemical resistance, while particles of silicon carbide are a reinforcement phase to significantly improve wear resistance, toughness, thermal conductivity, thermal shock resistance, and high-temperature creep resistance [106]. Compared to pure alumina, the wear resistance of Al_2_O_3_-SiC_w_ ceramic nanocomposites manufactured by spark plasma sintering was approximately two times higher, regardless of the applied load [107]. According to Raman spectroscopy, adding 0.5 vol.% of graphene nanoparticles primarily enhances wear resistance by forming a protective tribolayer. The tribolayer on the worn surfaces provides adequate lubrication, which reduces both the wear rate and the friction coefficient [107].

**Figure 11:**
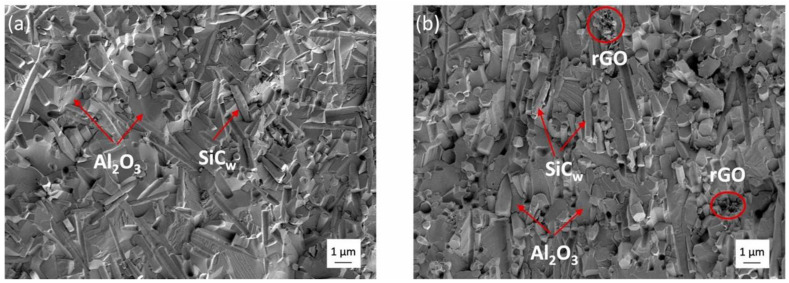
SEM micrographs of alumina reinforced with 17 vol.% SiC whiskers: (**a**) Without added graphene; (**b**) With 0.5 vol.% of reduced graphene oxide (Reproduced with permission from Suárez, M. et al., Ceramics International; published by Elsevier, 2024) [108].

A recent study further analyzed the influence of varying additions of 0, 0.5 vol.%, 1 vol.%, and 1.5 vol.% of reduced graphene oxide (rGO) to alumina-based, 17 vol.% silicon carbide whisker-reinforced ceramic composite (Al_2_O_3_-SiC_w_-rGO), manufactured by spark plasma sintering (SPS) [108]. Alumina grain size decreased with increasing rGO content (Figure 11). It was observed that even minimal quantities of reduced graphene oxide significantly alter the interfacial energy of Al_2_O_3_-SiC_w_ by diminishing the amount of intragranular SiC_w_. The composite with 0.5 vol.% graphene exhibited superior mechanical properties and exceptional scratch resistance compared to composites with higher graphene content and those without graphene added (Table 3).

### 2.8. Other Composite Alumina Ceramics

Ceramic composites of alumina and silicon carbide (SiC) were produced through powder metallurgy at a temperature of 1600 °C and a pressure of 30 MPa for 120 min. The findings indicate that the average grain size of the alumina matrix reduces as the SiC particle content rises. Maximum strength (1237 MPa) and toughness (5.68 MPa·√m) were obtained with 15 vol.% SiC content [109].

The study by Grigoriev et al. focused on creating novel electrically conductive ceramics: alumina composites with 30 and 40 vol.% titanium carbide (TiC) [110]. These composites are suggested for manufacturing cutting inserts for machining superhard steels, challenging materials, composites, etc. Composites produced by spark plasma sintering demonstrate adequate electrical conductivity, allowing them to be used in electrical discharge machining [110].

Alumina matrix composites reinforced with graphene oxide (GO) were manufactured through Spark Plasma Sintering. The findings indicate an approximately 35% enhancement in fracture toughness for composites with 0.5 wt.% GO compared to sintered pure alumina. SEM/TEM micrographs revealed a strong interface between the reinforcement and the matrix, and mechanisms such as branching, deflection, and bridging of crack propagation [111].

In a different study, graphene oxide was applied to silicon nitride (Si_3_N_4_) through electrostatic self-assembly processing. The Si_3_N_4_-GO powders were added to Al_2_O_3_/WB_2_ ceramic, which is sometimes used for precision cutting tools. the best mechanical properties were achieved at 7.94 vol.%. Compared to the Al_2_O_3_/WB_2_ ceramic tool material, this resulted in an increase of 30.3% in flexural strength and 36.7% in fracture toughness [112].

Similarly, silicon carbide (SiC) nanoparticles were coated with graphene (SiC-G). These were then incorporated into alumina as the matrix and sintered using spark plasma. The SiC-G nanoparticles achieved a uniform dispersion within the alumina-based composite for the ceramic tool, leading to stronger bonds between the alumina grains. The contact area between the graphene and the matrix was enhanced by the development of a 3D graphene mesh, which impeded the sliding of the graphene. The fracture toughness increased by 75.6%, and the flexural strength improved by 28.7%. When the composite was tested as a tool for 40Cr (equivalent steel grade ISO 41Cr4) hardened steel, the results demonstrated that adding SiC-G nanoparticles enhances the cutting life by 18.1%, while reducing the cutting force by 6.3% and the friction coefficient by 14.8% [113].

## 3. Advanced Sintering Techniques

Ceramics can only achieve the required density through sintering, which involves prolonged heating at high temperatures. In recent decades, significant attention has been dedicated to developing new sintering techniques. Alongside improving ceramic properties, reducing sintering time, temperature, and energy usage is crucial when assessing the environmental impact. Several methods are being studied, typically involving various additives in different amounts while applying modified or novel techniques and optimizing process parameters.

### 3.1. Two-Step Sintering Using Nanoparticles

Processes that ensure effective, consistent, and uniform distribution of nanoparticles within the primary ceramic matrix are being explored. Higher heating rates slow down grain growth in the conventional sintering of nano-alumina. Two-step sintering is an established method that inhibits grain growth even more effectively. The underlying mechanisms are still insufficiently understood. A sharp increase in the activation energy of grain boundary mobility at low temperatures could be the cause [114]. Comparing the alumina produced through two-step and conventional sintering reveals variations in density, grain size, and mechanical properties. Despite having a lower relative density of 97.64 from two-step sintering, compared to 98.2% from conventional sintering, the mechanical properties have shown improvement: flexural strength from 286 MPa to 303 MPa, fracture toughness from 4.09 MPa·√m to 4.35 MPa·√m, and Knoop microhardness from 17.5 GPa to 17.6 GPa [115].

### 3.2. Spark Plasma Sintering Using Co-Precipitated Powders

**Figure 12:**
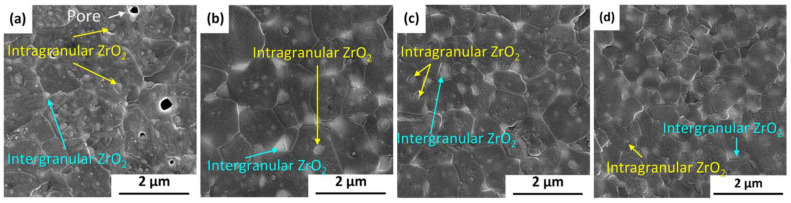
SEM micrographs of ZTA ceramics sintered from powders calcinated at different temperatures with different phase compositions: (**a**) 700 °C; (**b**) 900 °C; (**c**) 1100 °C; (**d**) 1300 °C (Adapted and reproduced with permission from Li, J. et al., Materials; published by MDPI, 2024) [116].

Li et al. [116] recently studied ZTA spark plasma sintering (SPS) using co-precipitated powders with varied phase compositions [116]. Results indicate that ZTA ceramics with an intragranular structure are more easily fabricated when composite powders with amorphous or transition phase Al_2_O_3_ as starting materials are used. Calcination of powders at different temperatures (700 °C, 900 °C, 1100 °C, and 1300 °C) showed that the best spherical shape and best dispersion were obtained after calcination at 1100 °C, as determined by scanning electron microscopy (Figure 12).

One other study has dealt with nano-ZrO_2_ adsorbed on the surface of the ZTA matrix. Zirconia particles can penetrate the interior of matrix grains due to the strong electric field generated by spark plasma sintering [117]. With a rapid heating rate of 100 °C·min^−1^ during SPS, low temperatures, and high currents, transporting substances quickly facilitates effective grain growth [118].

Spark plasma sintering of titanium carbonitride-reinforced ZTA ceramics at 1600 °C to 1675 °C was compared to pressureless sintering at 1720 °C with different carbon-to-nitrogen ratios in titanium carbonitride nanosized powder. The use of Ti(C_0.5_,N_0.5_) most significantly increased the Vickers hardness (over 19.0 GPa), while the apparent density was not significantly influenced [119].

### 3.3. Cold Sintering

The cold sintering process (CSP) is promising for exploring structural and mechanical properties and how they can be adjusted by changing the sintering conditions. Typically, CSP involves the uniaxial pressing of oxide powders at pressures ranging from 50 MPa to 500 MPa, along with the presence of a liquid and temperature kept below 350 °C. Applying this manufacturing technique to alumina ceramics is attractive and challenging and is currently the focus of a limited number of recent studies [120,121].

### 3.4. Low Temperature Sintering

**Figure 13:**
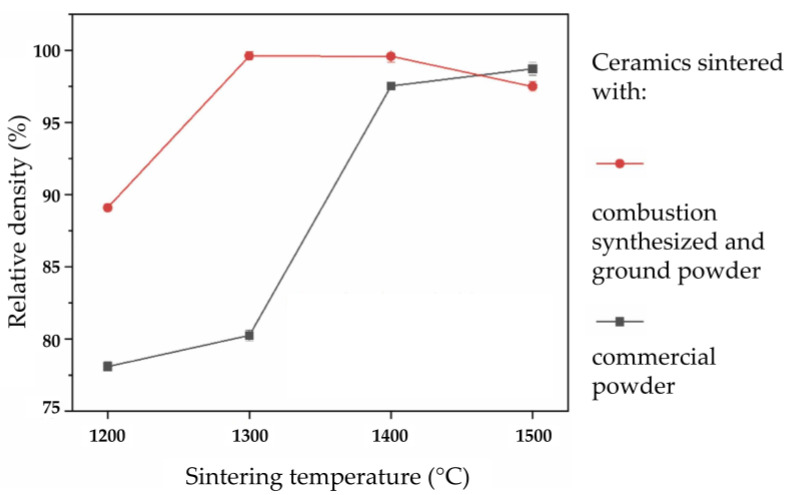
The relative densities of the sintered alumina as a function of sintering temperature (adapted and reproduced with permission from Xudong, L. et al., Journal of Alloys and Compounds; published by Elsevier, 2024) [122].

Due to strong bonding effects, sintering ceramics typically requires high processing temperatures above 1500 °C to obtain the required density. Compared to metals with metallic bonds, ceramics bond by covalent or ionic bonds, resulting in higher melting temperatures and energy requirements [122]. High sintering temperatures are typically associated with grain growth and lowering of the sintered ceramic’s mechanical properties. Nanoparticles and specific oxides enable the sintering of alumina at lower temperatures, within the range of 1300 °C to 1400 °C. Xudong et al. [122] studied in 2024 the sintering of ZTA at lower temperatures (1300 °C) using hot press sintering with ultra-fine (~5 nm) Al_2_O_3_ and ZrO_2_ nanoparticles [122]. By lowering the sintering temperature by 200 °C relative to what is needed for commercially available alumina powder, the nanoparticle sizes ranged from about 50 nm to less than 5 nm, resulting in a high relative density (Figure 13).

**Figure 14:**
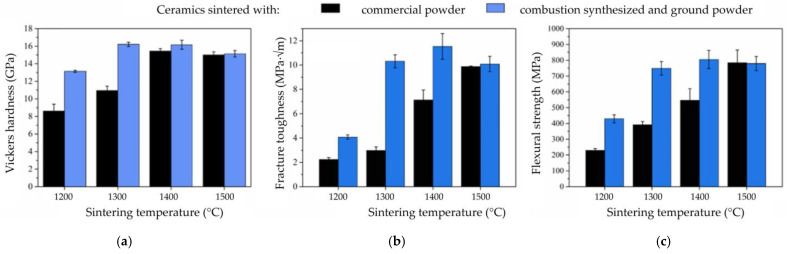
Low-temperature sintered ZTA ceramics: (**a**) Vickers hardness; (**b**) Fracture toughness; (**c**) Flexural strength (adapted and reproduced with permission from Xudong, L. et al., Journal of Alloys and Compounds; published by Elsevier, 2024) [122].

The nanoparticles exhibit strong coherence with the matrix. This is attributed to the structural similarities between tetragonal zirconia and α-alumina. The presence of nanoparticles has changed the fracture mode from intergranular to transgranular due to the grain boundary strengthening effect. A significant enhancement of mechanical properties was observed: Vickers hardness of 16.2 GPa ± 0.24 GPa, fracture toughness of 10.3 MPa·√m ± 0.53 MPa·√m, and flexural strength of 749 MPa ± 43 MPa (Figure 14) [122].

### 3.5. Microwave Sintering Using Nanoparticles

One innovative technique is microwave (MW) sintering, in which energy transfer occurs at the molecular level as the material absorbs microwave energy. In this manner, heat is generated within the material and on its surface, allowing for uniform heating. Microwave heating is faster than conventional heating, too. This limits the grain growth and the coarsening of the microstructure, which, in turn, improves the mechanical properties of the sintered ceramics [123].

In microwave sintering, materials are heated using an electromagnetic field when a dielectric material absorbs the energy throughout its volume and converts it into heat. The capacity of the material to heat up by microwaves is determined by its dielectric properties, whereby materials with a high dielectric loss factor heat up quickly. Alumina has a low dielectric loss factor, so it is almost transparent to microwaves. Therefore, an external susceptor is needed for its sintering [124]. A susceptor is a material—often silicon carbide—that effectively interacts with microwaves and transfers heat to the sample, mostly through radiation. On the other hand, tetragonal zirconia polycrystal (TZP), depending on the phase, dopants, and temperature, has a good or excellent dielectric loss factor, so when combined with alumina in ZTA, it allows heating by microwaves.

The effectiveness of alumina in MW sintering relies on the type of zirconia used: a study was recently published on the comparison of four different ZTA ceramics microwave sintered without the susceptor: unstabilized ZTA and ZTA with zirconia stabilized by 10 mol.% ceria were difficult to sinter, and slightly better densification was achieved by 3 mol.% yttria-stabilized ZTA. In contrast, the best results were obtained when zirconia was stabilized by 8 mol.% yttria. Yttria generates excess O^2−^ vacancies in the crystalline lattice of zirconia by doping it with Y^3+^, i.e., the lattice with a higher number of dipoles would explain better coupling of such ceramics [124].

Du et al. [125] examined 3 vol.% ZTA composite ceramics prepared through grinding and dispersing processing. The ceramics were microwave sintered at 1500 °C for 30 min [125]. Numerous nano-sized intragranular zirconia particles were dispersed within the alumina grains. A superlattice structure with a coherent link between the two phases was formed. This resulted in enhanced mechanical properties: flexural strength was 520.21 MPa, fracture toughness was 7.64 MPa·√m, Vickers hardness was 18.79 GPa, and the relative density was high at 99.07% [125].

### 3.6. Electron Beam Powder Bed Sintering

Zirconia-toughened alumina with functionally gradient properties is difficult to manufacture by sintering a multilayer ceramic preform (so-called green body). Rather, 3D rapid prototyping and manufacturing processes are being studied in sintering ceramics layer-by-layer using laser or electron beam. Considering the optical properties of ceramics, specific issues arise and must be addressed when opting for laser sintering rather than electron beam. On the other hand, the use of electron beams is connected to the issues associated with certain ceramics having low dielectric loss factors. Both processes also address the uneven heat distribution and large thermal gradients that can lead to uneven grain size distribution and inconsistent density, which can cause cracking in the sintered material.

Electron beam powder bed fusion (EB-PBF) is commonly used in additive metal manufacturing. In a 2024 study by Sjöström et al. [126], the surface of pre-sintered alumina was treated with an electron beam to induce a 1 µm nickel layer to increase the surface electrical conductivity and reduce smoke formation during electron beam sintering [126]. The nickel-coated alumina powder was effectively melted, demonstrating encouraging outcomes for its application in additive manufacturing. Issues such as irregular melting tracks, hole creation, and higher cracking were reported.

A preliminary study was recently conducted to enhance thermal uniformity by gradually controlling the process parameters during the sintering of 3 mm thick ceramic samples consisting of four layers with different properties and compositions: 100 wt.% alumina; 70 wt.% alumina and 30 wt.% zirconia; 30 wt.% alumina and 70 wt.% zirconia, and 100 wt.% zirconia. The electron energy was slowly raised from 2 eV to 7 keV, as was the beam current from 18 mA to 25 mA. The electron beam power change rate was restricted to 5 W/min in the early stages of heating. As the sample surface temperature reached 1000 °C, the electron beam power change rate was raised to 10 W·min^−1^. After 20 min were needed to reach 1400 °C, the surface temperature was maintained at this level for five more minutes. After that, the electron beam power change rate was slowly reduced to 5 W·min^−1^. Once the surface temperature was down to 500 °C, the electron beam was switched off, allowing the sample to cool in the vacuum chamber for 30 min. Samples formed before sintering with uniform layer thicknesses resulted in cracks and delamination, unlike those that did not have an even layer thickness. This was due to the differences in the thermal conductivities of the two materials used, alumina and zirconia, at room temperature of 20 W·m^−1^·K^−1^ and 1.8 W·m^−1^·K^−1^, respectively. To minimize the risk of cracking during electron beam sintering, the layers of materials with higher thermal conductivity should be made thinner compared to materials with lower conductivity [127].

## 4. Discussion

This overview of recent studies in the expansive domain of alumina-based cutting inserts shows that this research field is quite extensive, encompassing various areas of interest for scientists. The previous chapter provides an overview of studies on applying different sintering methods for alumina cutting inserts. When sintering alumina for use as cutting inserts, the primary goal is to maximize density and toughness while keeping a low grain size. Various unconventional sintering methods and their modifications are currently utilized to optimize properties. Typically, the effect of sintering method and process parameters varies across different studies. Numerous impacts on final properties occur before the sintering process. To start with, various manufacturers produce alumina powder of different purities. Powders vary in average particle size and particle size distribution. The use of various additives in differing quantities is also a commonplace: Binders are utilized to enhance the strength of the ceramic green body, that is, the body before it undergoes sintering; Plasticizers reduce green body brittleness; Dispersants facilitate a stable dispersion when the green body is formed through slip casting; Lubricants are used to reduce friction when a green body is made by pressing, etc. Additionally, the influence of various equipment used must be considered, as discrepancies in manufacturers, models, or configurations can significantly affect deagglomeration, potential contamination from the jar or balls in ball-milling, precision in uniform pressing, and the accuracy of temperature, time, voltage, and microwave exposure control during the sintering process. Consequently, comparing the key mechanical properties of pure alumina ceramics sintered in different ways is only approximately feasible (Table 4). It is also important to note that studies aimed at improving the properties of alumina are typically not conducted in its unaltered state; rather, they incorporate additional chemical compounds, which may include dopants (typically below 5 wt.%) or second-phase ceramics, as well as various novel nanoparticles.

However, alumina-based cutting inserts constitute a relatively small portion of the diverse materials within the broader domain of cutting inserts. In addition to advanced technical ceramic cutting inserts, cemented carbide remains today’s most prevalent material. High-speed steels are also utilized. Ceramics commonly used for machining include various materials: alumina-based with different additions, silicon nitride-based, polycrystalline cubic boron nitride, and polycrystalline diamond (the famous “superhard tool”). Different materials utilized for cutting inserts facilitate the fulfillment of various application requirements, each exhibiting distinct strengths and weaknesses (Table 5).

Alumina-based cutting inserts are used in machining when needed due to their unique properties: high hardness, wear resistance, and thermal stability. Nonetheless, these tools have limitations and challenges, restricting their use in more specialized applications. Addressing these properties continuously through intensive research makes ceramic cutting inserts more accepted in machining.

As this paper outlined, current research focuses on numerous improvements to alumina-based cutting inserts and their modifications. This indicates numerous potential ways in which future ceramic-based cutting materials will be enhanced and new market improvements will be achieved. Advanced technical ceramics and ceramic matrix composites that exhibit enhanced fracture toughness, while maintaining elevated hardness levels, will facilitate an increase in dry machining and high-speed finishing. Continuous improvement and dependable properties yield economic benefits for companies in the tooling and manufacturing sectors, justifying the implementation of advanced ceramics in industrial settings by reducing downtime and tool failure and improving process efficiency, allowing for higher cutting speeds and durability. When addressing alloys that are difficult to machine, such as heat-resistant steels, titanium alloys, or nickel- and cobalt-based superalloys, continuous development in the sector of ceramic-based cutting inserts will provide significant industrial advantages. Developing new hardened steels and superalloys, featuring intermetallics or hard carbides, as well as new metal-matrix composites, is linked to an increasing future need for advanced ceramic cutting inserts.

## 5. Conclusions, Sustainability and Perspective

The areas of advancement in ceramic cutting inserts are vast, as this paper briefly covers, focusing only on alumina-based ceramic cutting inserts. Adding other chemical elements or compounds to control and modify the crystal structure, tailoring the properties, managing the grain size, porosity, and density, adjusting thermal and electrical conductivity, susceptibility to microwave heating, and so on, opens endless possibilities in optimizing their properties and allowing for improved wear, adhesion, and chipping and fracture resistance of ceramic cutting inserts.

Several significant environmental and societal concerns regarding sustainability are associated with commonly used cemented carbide cutting inserts, related to cobalt, which acts as a binder metal. Cobalt and tungsten are additionally identified as critical raw materials. Substituting cemented carbide with ceramics is advantageous in its own right.

Utilizing ceramic cutting inserts in dry machining offers a sustainable alternative. It enhances workplace safety, as liquid coolants usually contain mineral oils and organic additives such as lubricants, emulsifiers, and corrosion inhibitors. Additionally, the waste management of cutting fluids entails high costs and long-term environmental hazards. The potential of MWCNT-alumina composite cutting inserts also reduces the reliance on liquid coolants.

Promoting more sustainable economic growth related to machining processes can be enhanced by extending the tool life of ceramic cutting inserts, which decreases the raw materials required for tool production and conserves the energy needed for sintering.

The various perspectives for enhancing the properties and expanding the applications of ceramics-based cutting inserts, including those alumina-based ones, appear to be promising soon.

## Figures and Tables

**Table 1:** Properties of Al_2_O_3_ compared to ZrO_2_ and Si_3_N_4_ (data from Volosova, M.A. et al., Technologies (Basel); published by MDPI, 2020) [36]).

	Density (g·cm^−3^)	Melting Point (°C)	Flexural Strength (MPa)	Vickers Hardness (GPa)	Fracture Toughness (MPa·√m)	Thermal Conductivity (W·m^−1^·K^−1^)	Thermal Expansion Coefficient (10^−6^·K^−1^)
Al_2_O_3_	3.8–4.0	2044	300–350	19–21	3.0–3.5	25–30	8.0–9.0
ZrO_2_	6.0–6.05	2715	750–1050	12–13	8.0–10.0	2–3	10.0–11.0
Si_3_N_4_	2.37–3.25	1900	650–800	16–19.6	6.5–7.2	10–43	1.4–3.7

**Table 2:** Properties of SrCO_3_ modified ZTA (Data selected and reproduced with permission from Thakur, T. et al., Heliyon; published by Cell Press, 2025) [6]).

Content of SrAl_12_O_19_	0 wt.%	2.5 wt.%	5.0 wt.%	7.5 wt.%
Density after HIP (%)	100	99.77	100	99.76
Density after Sintering (%)	99.77	99.53	99.77	99.29
Young’s Modulus (GPa)	362.7	358.44	362.64	353.45
Vickers Hardness (GPa)	19.85	20.12	21.04	18.53

**Table 3:** Mechanical properties of alumina-based composite reinforced with SiC whiskers and with added 0.5 vol.% graphene (selected and reproduced with permission from Smirnov, A. et al., Nanomaterials; published by MDPI, 2019, and Suárez, M. et al., Ceramics International; published by Elsevier, 2024) [107,108].

Composition	Flexural Strength (MPa)	Fracture Toughness (MPa·√m)	Hardness * (GPa)
Al_2_O_3_	750	9.5	10.6
Al_2_O_3_-17%SiC_w_	750	9.52	28.8
Al_2_O_3_-17%SiC_w_/0.5%Graphene	919	10.55	29.9

* Additional properties available in [108].

**Table 4:** An approximate comparison of pure alumina properties sintered by different methods.

Sintering Process	Temperature (°C)	Flexural Strength (MPa)	Hardness (GPa)	Young’s Modulus (GPa)	Fracture Toughness (MPa·√m)	Lit. Ref.
Conventional	1650	353	16.8	323	2.6–2.8	[128,129]
Two-step	1550; 1450	303	17.5	303	4.35	[115]
Spark plasma	1500	710	21.3	-	2.9	[130]
Thermal- assisted cold	1350	-	14	335	-	[122]
Microwave	1500	-	16.19	365.4	2.7	[131]

**Table 5:** A comprehensive overview of the pros and cons of ceramic cutting tool materials and their applications [83,112,132,133,134,135,136,137,138,139].

Material	Benefits *	Drawbacks *	Applications
Al_2_O_3_	High hardness and wear resistance	Low fracture toughness and thermal shock resistance	High-speed cutting of cast iron and steel
Al_2_O_3_ + ZrO_2_	Higher thermal stability, higher toughness	Increased price	Interrupted cutting
Al_2_O_3_ + ZrO_2_ + MgO	MgO (0.2 wt.%) increases dynamic compressive strength	Too much MgO decreases dynamic compressive strength	Higher cutting-edge stability
Al_2_O_3_ + ZrO_2_ + Mo	Higher electrical conductivity	Reduced oxidation resistance at high temperatures	Tool shaping by EDM (electrical discharge machining)
Al_2_O_3_ + MnO	Less porosity, higher wear resistance, and compressive strength	Decrease in Young’s modulus	Finishing operations of hardened steels
Al_2_O_3_ + TiO_2_	Less porosity, higher wear resistance, and toughness	Decrease in bending strength if more than 0.5% TiO_2_	Cutting hard or abrasive materials
Al_2_O_3_ + ZrO_2_ + SrCO_3_	Finer and more uniform grain structure	Too much SrCO_3_: formation of secondary phase or porosity	Tools with tailored dielectric properties
Al_2_O_3_ + WB_2_	Much higher hardness and melting point	Lower toughness and thermal shock resistance	Precision cutting tools
Al_2_O_3_ + ZrO_2_ + MgO + MWCNTs	Significant improvement in wear resistance	Difficult processing due to MWCNTs	Low feed rates and shallow cutting depths
Al_2_O_3_ + SiC_w_	Improved toughness compared to Al_2_O_3_	Difficult to manufacture and more expensive	Superalloys cutting
Al_2_O_3_ + SiC_w_ + GO	GO improves toughness, thermal and electric conductivity	Difficult processing due to GO	Intermittent cutting, tool shaping by EDM
Cemented carbide (WC + Co)	Lower hardness, more versatile, lower cost	Lower chemical stability, shorter tool life due to lower hardness	Wet and dry cutting, various materials
Cermets (no WC + Co)	Sharper edges, good crater wear resistance, lower cost	Lower hardness, i.e., shorter tool life	Good for finishing
Si_3_N_4_	Good thermal shock resistance and toughness	Lower hardness, more expensive	Intermittent cutting, superalloys
PcBN	Extreme hardness, long tool life	Difficult to machine, expensive	Machining hard and abrasive materials, hard turning
PCD	Extreme hardness, long tool life	Reacts with iron at high temperatures	Machining aluminum alloys, MMC, and CFRPs

* All properties compared to Al_2_O_3._

## Data Availability

No new data were created or analyzed in this study. Data sharing is not applicable to this article.

## Abbreviations

The following abbreviations are used in this manuscript:

3Y-TZP	3% Yttria-Tetragonal Zirconia Polycrystal
CFRP	Carbon Fiber Reinforced Composites
CMC	Ceramic Matrix Composites
CRM	Critical Raw Materials
CSP	Cold Sintering Process
EB-PBF	Electron Beam Powder Bed Fusion
EBM	Electron Beam Melting
EDM	Electrical Discharge Machining
FESEM	Field Emission Scanning Electron Microscopy
GO	Graphene Oxide
MMC	Metal Matrix Composites
MQL	Minimum Quantity Lubrication
MW	Microwave
MWCNT	Multi-Walled Carbon Nanotubes
PcBN	Polycrystalline Cubic Boron Nitride
rGO	Reduced Graphene Oxide
PCD	Polycrystalline Diamond
SDG	Sustainable Development Goals
SEM	Scanning Electron Microscopy
SiC_w_	Silicon Carbide Whiskers
SPS	Spark Plasma Sintering
TEM	Transmission Electron Microscopy
TZP	Tetragonal Zirconia Polycrystal
YSZ	Yttria-Stabilized Zirconia
ZTA	Zirconia-Toughened Alumina
